# The Influence of Non-Engineered Municipal Landfills on Groundwater Chemistry and Quality in Bloemfontein, South Africa

**DOI:** 10.3390/molecules25235599

**Published:** 2020-11-28

**Authors:** Rinae Makhadi, Saheed A. Oke, Olusola O. Ololade

**Affiliations:** 1 Department of Geology, University of the Free State, Bloemfontein 9301, South Africa; MakhadiR@ufs.ac.za; 2 Centre for Environmental Management, University of the Free State, Bloemfontein 9301, South Africa; shola.ololade@gmail.com; 3 Civil Engineering Department, Central University of Technology, Bloemfontein 9300, South Africa

**Keywords:** borehole, geology, groundwater, landfill, SAR, water quality

## Abstract

This study assessed the groundwater quality around two municipal solid waste landfill sites, in the city of Bloemfontein, Free State Province, South Africa. The two landfill sites are located in two contrasting geological terrains, with both lacking some basic facilities found in a well-designed landfill. A total of eight groundwater samples were collected from pollution monitoring boreholes near the two landfill sites, with five samples representing the northern landfill site and three samples representing the southern landfill site. The samples were collected in the autumn and winter seasons to assess any possible seasonal variations. They were analysed for physicochemical (pH, electrical conductivity (EC), total dissolve solids (TDS), chemical oxygen demand (COD) and total organic carbon (TOC)) and microbiological parameters (*Escherichia coli*, total coliform). The results of the analysis showed that the waters from both landfills were generally dominated by Ca, Mg, SO4, and HCO3 ions. Some of the major anions and cations in the water samples were above the South African National Standard (SANS241:2015) and World Health Organisation (WHO) permissible limits for drinking water. Majority of the boreholes had total dissolved solids and electrical conductivity values exceeding the SANS 241:2015 and WHO permissible limits. Piper trilinear plots for the two landfill sites showed that Ca(Mg)HCO3 water type predominates, but Ca(Mg)SO4 and Ca(Mg)Cl were also found. These water types were further confirmed with expanded Durov diagrams, indicating that that the boreholes represented a water type that is seldom found which is undergoing ion exchange, typical of sulphate contamination. From the SAR diagrams, boreholes in the northern landfill site had a high salinity hazard with only one borehole in the southern landfill site having a high salinity hazard. The geology was found to play a significant role in the distribution of contaminants into the groundwater systems in the study area. The study concluded that the northern landfill site had a poorer water quality in comparison to the southern landfill site based on the analysed physicochemical parameters. However, the southern landfill site showed significant microbial contamination, due to the elevated amount of *E. coli* and total coliform concentrations. The high permeability of the weathered dolerites in the northern landfill site might have enabled the percolation of contaminants into the groundwater resulting in the poorer water quality.

## 1. Introduction

Water is regarded as an essential requirement of life which has been considered more as an economic resource than a social good [1]. According to the National Water Act (Act No. 36 of 1998), water is fundamental for all life, and no person, plant, animal or living organism can survive without water. An increase in population, urbanisation, irrigation and domestic activities have resulted in the overexploitation of available water resources of which surface water has been the most reliable source of water [1]. According to Pietersen et al. [2], surface water resources in South Africa have over the years been unable to meet the country’s water demands, and groundwater has become a potential and convenient source of drinking water. Groundwater is considered as a safe source of drinking water because it is abstracted with low microbial load, and minimal treatment is required before consumption [3]. Unfortunately, groundwater resources are commonly vulnerable to pollution, most especially in the present day wherein extreme contamination takes place on the surface, and eventually reaches the groundwater systems. The contamination of groundwater resources either by natural or anthropogenic sources, ultimately degrades their quality rendering it unfit for use.

The disposal of waste by landfilling has over the years proven to be one of the main culprits behind the contamination of groundwater resources [4]. According to Naveen et al. [5], municipal solid waste landfills create a lot of pollution, due to the leakage of landfill leachate that affects the surrounding environment, especially surface and groundwater bodies. Nagarajan et al. [6] further illustrate that areas near landfills have a greater possibility of groundwater contamination because of the potential pollution source of leachate originating from the nearby dumping site. Findings from Singh and Mittal [7], stipulate that the leachate generated from landfills contain excess contaminants which even at trace levels can deteriorate the overall quality of water, as well as cause severe health problems. According to Bjerg et al. [8], the anaerobic leachate that develops in landfills comprises of high content of dissolved organic carbon, salts, ammonium and organic compounds, as well as metals.

There are a plethora of factors that need to be considered when selecting a site that will be suitable for locating a landfill site. Allistar and MacCarthy [9], suggests that the geology of an area with an emphasis on the underlying bedrock and geological structures is one of the most important factors that must be considered when determining the suitability of a site for landfill construction. Hydrogeological, topographical, ecological and economic, as well as the social environment are some of the main factors that influence the suitability of waste disposal sites [9]. From a geological/hydrogeological perspective, a variety of factors have to be considered to minimise and prevent the contamination of groundwater resources, and these include: The bedrock lithology, quaternary geology, hydrological properties, geological structures, hydrogeology and the topography of an area [9]. 

Bloemfontein, a metropolitan city within the Free State Province of South Africa with an increasing population associated with people migrating from rural areas, resulting in an increase in a waste generation [10]. Groundwater is currently not used as a source of potable water supply in Bloemfontein, but it is used for irrigation in residential areas and extensively for agricultural purposes in Bainsvlei and areas southwest of Bloemfontein [10]. Presently, there are two landfills in the city, one situated north and the other south of the city, and these sites are the major recipients of all the waste produced in the city [11]. According to the Mangaung Metropolitan Municipality [11], the two landfill sites are currently permitted to be used, but do not meet the minimum requirements as they lack some of the basic facilities required of a well-designed landfill site. These facilities include an underling barrier and leachate collection systems, as well as proper controls with regards to the health and safety of the reclaimers [11]. The two landfill sites are situated in two different geological terrains, although they are both underlain by Karoo sediments, slight differences do exist in the underlying geology [12].

In this study, a comparison of the groundwater quality surrounding the two municipal landfill sites was conducted. It is important to investigate the impact of the leachate emanating from the unlined landfill on the groundwater chemistry. Physicochemical and microbiological parameters of the groundwater samples from the vicinity of the two landfill sites were analysed to assess the impact of leachate on the groundwater quality and determine whether there are any significant differences in the water chemistry obtained from the two landfill sites. 

## 2. Materials and Methods

### 2.1. Study Area

**Figure 1:**
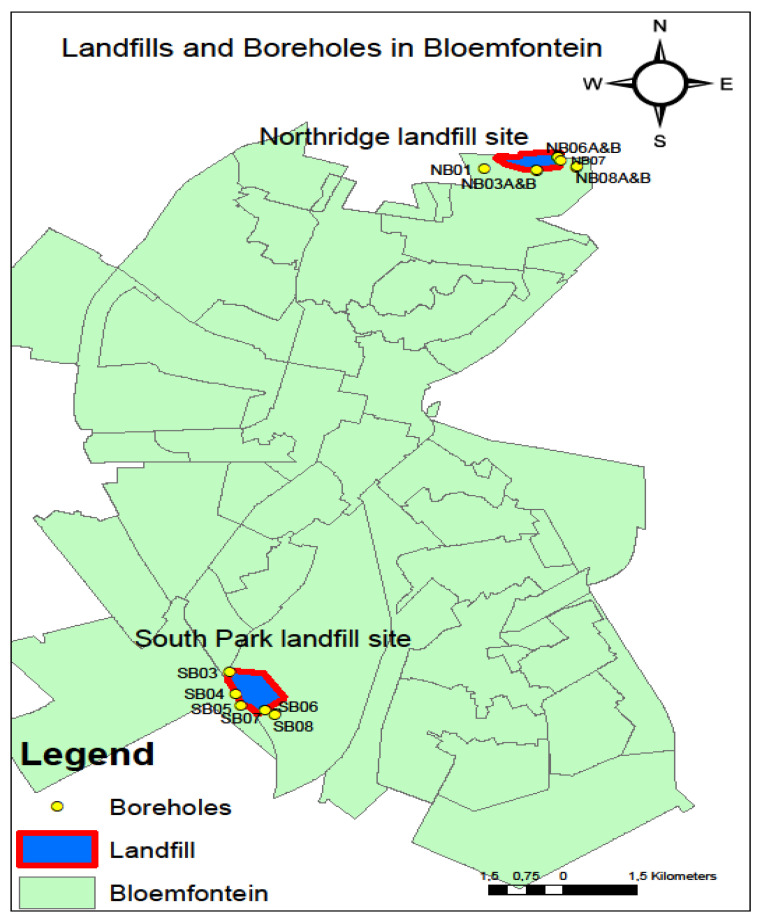
Map illustrating the two landfill sites in Bloemfontein and the location of the pollution monitoring boreholes in the vicinity of the two landfill sites.

The study area (Figure 1) comprises of two locations, namely, the northern landfill site and the southern landfill site, in Bloemfontein, Free State Province, South Africa. According to Dingaan and Du Preez [13], the climate of the area is categorised as a cold semi-arid climate (BSK) zone, which entails a steppe climate with dry winters and mean annual temperatures below 18 °C. The average annual precipitation in Bloemfontein is 514 mm, with an evaporation rate of 1676 mm [10]. The summer season ranges from October to February and winter from May to August, with an average annual mean maximum temperature ranging from 26 °C in summer and an average annual mean minimum temperature of 8 °C in winter [11]. The soil type of the study area was classified as a duplex soil, with a major portion being clay [14]. The geohydrological characteristics of the two areas under investigation are slightly different, as discussed in the next two sub sections. 

#### 2.1.1. The Geology (Rock Types and Hydrogeology) of the Northern Landfill Site

**Figure 2:**
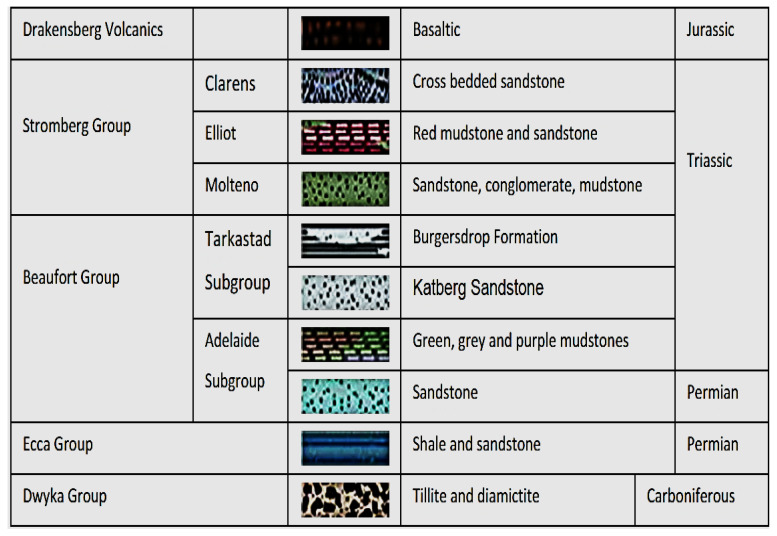
Schematic presentation of the Karoo Supergroup sequence [15].

The Bloemfontein northern landfill site is located in the northern margin of the city and is about 35 hectares in extent [12]. The landfill site is classified as a general waste site [11]. The landfill area is characterised by two geological groups of the Karoo Supergroup, namely, the Ecca and the Beaufort groups [11]. The Beaufort group consists of vast sedimentary rocks, such as sandstone, shale, and mudstone (Figure 2), which are intruded by dolerite dykes and sills [15]. Two aquifer types are common to the Bloemfontein area, namely, fractured and intergranular aquifers [11]. Intergranular aquifers comprise of sedimentary rocks that have a series of dolerite intrusions [12]. The dolerite dykes have an average borehole yield of between 0.5 L/s and 5 L/s [12]. According to Butler [12], the landfill has a steep slope to the north and is situated on a slight rise, which is controlled by high resistance to weathering of the underlying dolerite. Waste rock piles from a “dolerite mine, located on the north-western boundary and upgradient of the landfill site may affect the chemistry of the ground and surface water, as a large percentage of run-off from the mine drains through a portion of the landfill” [12]. Pollution monitoring boreholes were dug in the eastern and southern sides of the landfill [11]. The boreholes were drilled into dolerite, to a depth of about 35 m as the landfill site is completely underlain by a dolerite sill [12]. The expected groundwater flow is in a south-easterly direction following the topography [12]. Given the position of the pollution monitoring boreholes with reference to the landfill site, any leachate that flows out of the landfill site would flow towards the boreholes with respect to the topography [16]. 

#### 2.1.2. The Geology (Rock Types and Hydrogeology) of the Southern Landfill Site

The Bloemfontein southern landfill site is located in the southern margin of the city and is about 44 hectares in extent [12]. The landfill site is mainly a general waste site with a small area to the east designated for hazardous medical waste [11]. The landfill site is underlain by Karoo sediments of the Beaufort group (Figure 2), with siltstones and lenses of shale and sandstones being the most dominant and a dolerite dyke that is situated about 15 m south-east of the landfill [12]. The landfill slopes about 25° to the north-west. The pollution monitoring boreholes were drilled, downgradient of the landfill site. The boreholes were entirely drilled through horizontally bedded siltstones interbedded with layers of clay, sandstone and shale [12]. The expected direction of groundwater flow is in a south-westerly direction, following the gentle surface gradient.

### 2.2. Groundwater Sampling

To study the impact of leachate from the unlined municipal landfill sites, groundwater samples from the pollution monitoring boreholes in the vicinity of the two landfill sites (northern and southern) were collected (Figure 1). Duplicate samples were collected in autumn (2 March 2018) and winter (19 June 2018). The sampling names followed the municipal labelling of the boreholes (Figure 1). Groundwater sampling followed the description in the American Public Health Association [17]. A total of five sampling points “boreholes” were sampled in the northern landfill site consisting of boreholes NB03A, NB03B, NB07, NB06A and NB06B. Although boreholes NB01, NB08A and NB08B are included in the map (Figure 1), they were not sampled for the study as NB01 was destroyed during the development of a residential area near the landfill site, and NB08A and NB08B are located within a private property where no access was granted for sampling purposes.

For the southern landfill site, a total of three sampling points “boreholes” consisting of boreholes SB04, SB08A and SB08B. The other monitoring boreholes (SB03, SB05, SB07 and SB08) in the southern landfill site that are indicated in the map were not sampled as they were either damaged or collapsed. The water samples collected for physiochemical analysis were contained in tight-capped polyethene bottles, while the ones for microbiological analysis were in tight-capped glass bottles to avoid any contamination. They were kept in a cooler box containing ice before being transferred to the laboratory on the same day of collection for analysis. Water quality analyses (physical, cations, trace elements, anions, and microbial) of the groundwater samples were carried out at the Institute for Groundwater Studies (University of the Free State) using ICP-MS, ion chromatography, and IDEXX (Colilert18) Quanti-TrayTM.

### 2.3. Hydrogeochemistry

Piper diagrams, expanded Durov diagrams and sodium adsorption ratio (SAR) diagrams were plotted using the WISH (Windows Interpretation for Hydrogeologists) version 3.02.191, a software program developed by Lukas Eelco from the Institute for Groundwater Studies, University of the Free State. This software has been used extensively by other researchers [18,19]. The SAR diagrams were plotted to describe the water based on sodium and salinity hazard. Although the eight monitoring boreholes are not irrigation wells, households in the vicinity of the landfill sites use groundwater for irrigation purposes. Piper and expanded Durov diagrams plot of chemical variables from eight boreholes, as well as the classification of the water types. The expanded Durovs were compared with the suggested compliance monitoring standards given in the Department of Water Affairs minimum requirements for monitoring at waste management facilities [20].

## 3. Results

### 3.1. Physicochemical Characteristics of the Groundwater Samples of the Northern and Southern Landfill Sites

The physicochemical and microbiological analyses of the water samples for autumn and winter for the two landfill sites are presented in Table 1. The average concentrations of both physicochemical and microbiological parameters for all the boreholes in the two landfill sites are indicated in Table 2. Heavy and trace metal results presented very low or undetectable concentrations except for lead and arsenic, which had concentrations above the SANS 241 standards in all the boreholes. The pH for the borehole water samples from the two landfill sites ranged between 7 and 8 (Table 1). This indicated that all the sampling sites had a pH within the slightly alkaline range. TDS concentrations varied across the two landfill sites with boreholes in the northern landfill site having the highest TDS concentrations over the two seasons (Table 2). 

Borehole samples from the northern landfill site had a mean TDS concentration of 2365 mg/L for the autumn samples and 1994.7 mg/L for the winter samples, with boreholes in the southern landfill site having mean TDS concentrations of 1005 mg/L for the autumn samples and 906 mg/L for the winter season, respectively (Table 2). Borehole NB07 had the highest TDS concentrations for the northern landfill site with borehole SB04 having the highest TDS concentrations for the southern landfill site over both seasons (Table 1). Electrical conductivity values were higher for boreholes in the northern landfill site in comparison to the southern landfill site. Borehole samples from the northern landfill site had a mean EC of 316 mS/m for the autumn samples and 292 mS/m for the winter samples, while borehole samples from the southern landfill site had a mean of 140 mS/m over both seasons (Table 2). 

Borehole NB07 had the highest EC for the northern landfill site, and borehole SB04 had the highest EC value for the southern landfill site. The mean TOC and COD concentrations were also higher for boreholes in the northern landfill site in comparison to those in the southern landfill site. Boreholes from the northern landfill site had mean TOC of 27 mg/L for the autumn samples and 24 mg/L for the winter samples, while the samples from the southern landfill site had a mean of 6.9 mg/L for the autumn samples and 7.5 mg/L for the winter samples. COD concentrations also showed a similar trend with the other physicochemical parameters, with boreholes samples in the northern landfill site having a mean concentration of 142 mg/L for the autumn samples and 101 mg/L for the winter samples. The boreholes samples in the southern landfill site had a mean concentration of 24 mg/L for the autumn samples and 34 mg/L for the winter samples.

### 3.2. Microbiological Characteristics of the Groundwater Samples from the Northern and Southern Landfill Sites

Total coliform bacteria are indicative of the presence of disease-causing organisms that may be present in the water body. The total coliform mean values for the northern landfill site were 1095 cfu/100 mL for the autumn samples and 928 cfu/100 mL for the winter samples (Table 2). In contrast to the physicochemical parameters, boreholes in the southern landfill site had higher total coliform and *Escherichia coli* concentrations in comparison to the northern landfill site (Table 2). Boreholes in the southern landfill site had a mean total coliform concentration of 1194.5 cfu/100 mL for the autumn samples and 1377 cfu/100 mL for the winter samples. While total coliform in borehole NB03B and NB07 were higher for the autumn season and NB03A and NB03B for the winter season in the northern landfill site. *E. coli* concentrations were also higher for boreholes samples in the southern landfill site with no *E. coli* detected for the boreholes samples in the northern landfill site (Table 1). Boreholes samples in the southern landfill site had a mean *E. coli* concentration of 103 cfu/100 mL for the autumn samples and 28 cfu/100 mL for the winter samples.

### 3.3. Hydrogeochemical Facies

**Figure 3:**
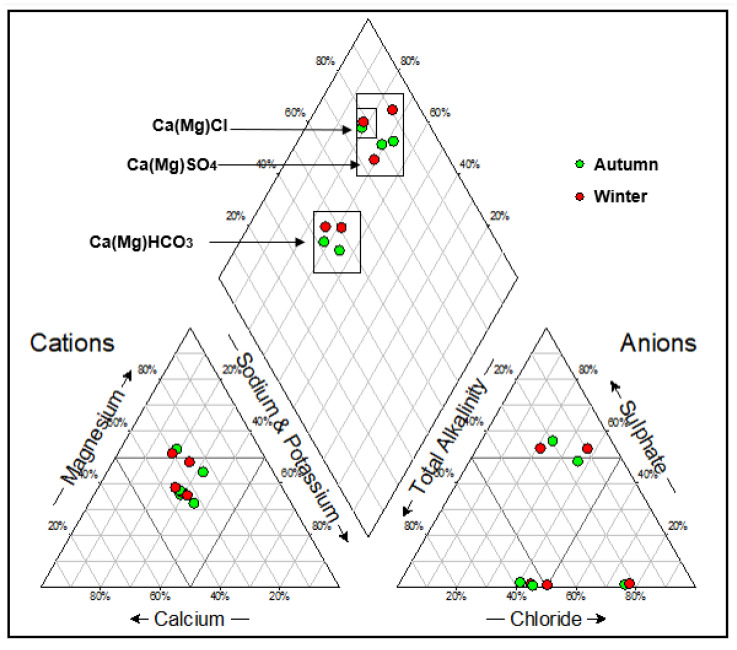
Piper diagram for the groundwater samples in the northern landfill site for the two seasons.

**Figure 4:**
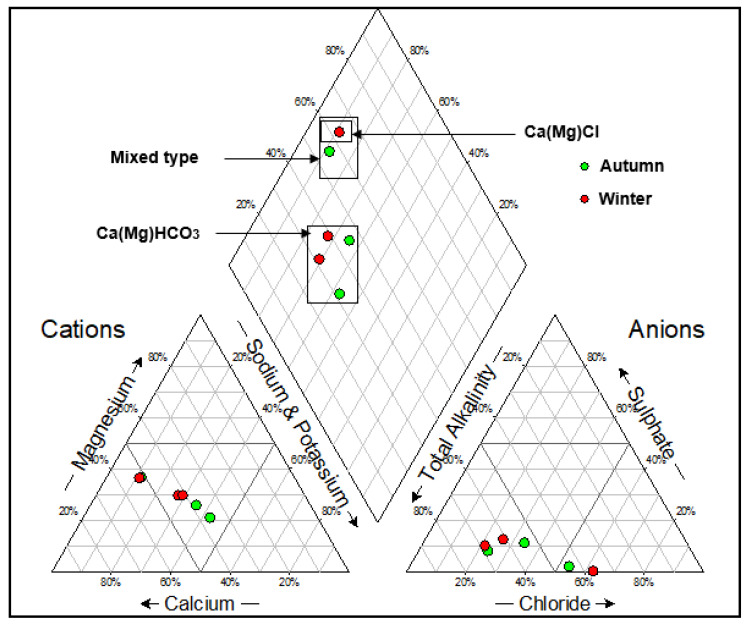
Piper diagram for the groundwater samples in the southern landfill site for the two seasons.

**Figure 5:**
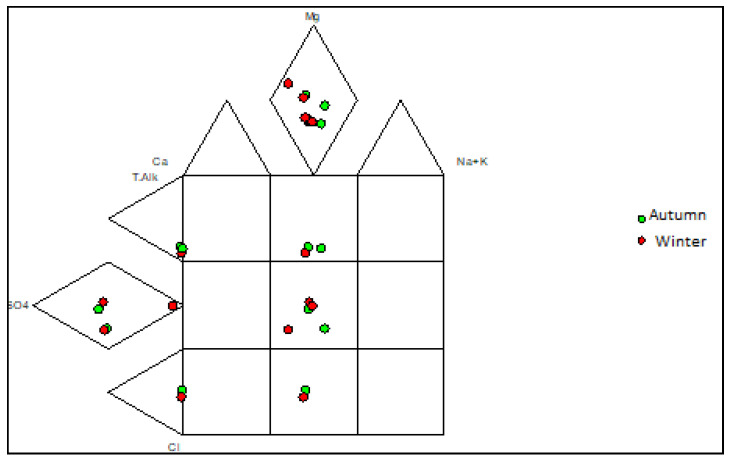
Expanded Durov diagram for groundwater samples in the northern landfill site for the two seasons.

**Figure 6:**
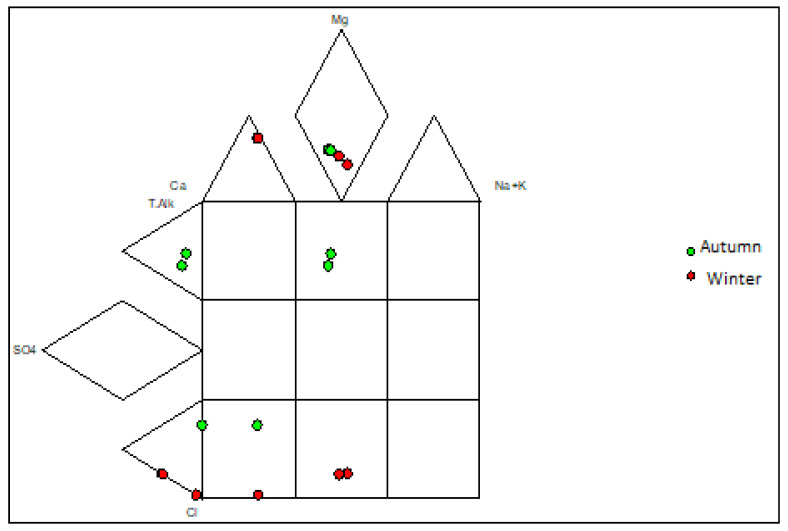
Expanded Durov diagram for groundwater samples in the southern landfill site for the two seasons.

Piper plots were constructed to illustrate the hydrogeochemical facies for the groundwater samples, and four main water types were identified (Figure 3 and Figure 4). Expanded Durov diagrams were plotted to further illustrate the eight chemical variables, as well as classify the water (Figure 5 and Figure 6).

The water samples from the two landfill sites showed variation in the overall major ion chemistry, as well as similarities. Boreholes NB06A and NB06B had similar chemistry and plotted in the Ca(Mg)HCO_3_ vicinity over both seasons. Boreholes SB08A and SB08B in the southern landfill site also plotted in the Ca(Mg)HCO_3_ vicinity over both seasons. These water samples are indicative of dominance in alkalinity and no dominant cation. Boreholes NB03A and NB03B also had similar chemistry and plotted in the Ca(Mg)SO_4_ vicinity in the two seasons. Borehole SB04 showed variation in the overall major ion chemistry over the two seasons. The borehole plotted in the mixed type vicinity in the autumn season and the Ca(Mg)Cl vicinity in the winter season. Borehole NB07 plotted in the Ca(Mg)Cl vicinity in the two seasons which indicated a dominance in the chloride anion and no dominant cation. 

**Figure 7:**
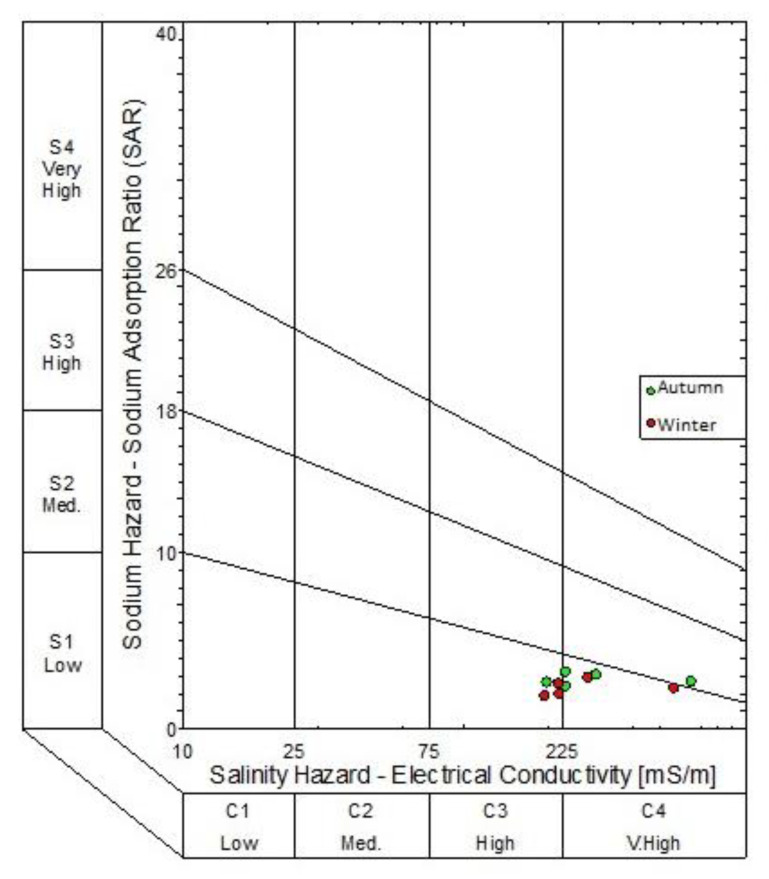
Salinity hazard (SAR) diagram for the boreholes in the northern landfill site.

**Figure 8:**
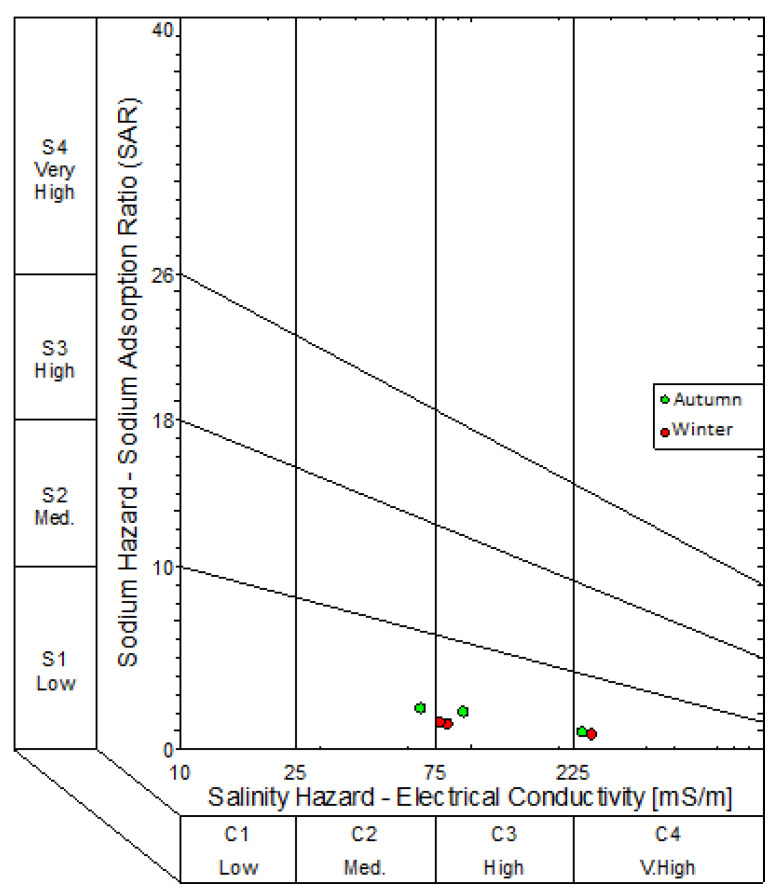
SAR diagram for the boreholes in the southern landfill site.

Results from the expanded Durovs shows the water samples from the northern landfill site plotted on the seldom found section, as well as the unpolluted water types based on minimum requirements suggested by the Department of Water Affairs requirements for water monitoring at waste management facilities. From a hydrogeochemical perspective, these water types are represented of water undergoing continuous changes in chemistry. Sodium Adsorption Ratio diagrams were constructed to illustrate the salinity hazard of the different groundwater samples based on their electrical conductivities (Figure 7 and Figure 8). The SAR diagram is plotted to illustrate the aquifers irrigation water quality within the vicinity of the landfill. In the northern landfill site, boreholes NB07 and NB03B had a very high salinity in both seasons. Borehole SB04 had a high salinity hazard over both seasons in the southern landfill site.

## 4. Discussion

### 4.1. Physicochemical Characteristics of the Groundwater Samples from the Northern and Southern Landfill Sites

All the groundwater samples had a pH within the permissible limits as prescribed by WHO [21] and SANS241 [22] for drinking water. The samples had pH values within the DWAF [23] specifications for irrigation. The WHO [21] and SANS241 [22] recommends that a TDS concentration below 500 mg/L and 1200 mg/L, respectively is suitable for drinking water. All the groundwater samples from the northern landfill site had TDS concentrations that exceeded both the SANS241 and WHO recommended limits for drinking water. All the groundwater samples in the southern landfill site had TDS concentrations that exceeded WHO drinking water limits, with only borehole SB04 exceeding the SANS 241 drinking water limits. According to Ngabirano et al. [24], high temperatures during dry seasons facilitate dissolution, ion-exchange capacity, desorption, and weathering processes. Considerable rainfall had been received prior to sampling in March 2018 after a long summer period where the study area had been relatively dry. This would have facilitated long-term dissolution, since the change in groundwater composition is not an instantaneous process, but occurs over time, thereby contributing to an increase in TDS [16].

Groundwater recharge through the dolerite dykes and fractures from rainfall that already contained elements in solution from the landfill could have facilitated more dissolution and caused considerable increases in TDS during autumn for the northern landfill site [12]. Regarding the EC values, mean EC values were much higher for boreholes in the northern landfill site in comparison to those in the southern landfill site. According to Kumar et al., [25] the EC of water samples is a useful tool in the assessment of the overall purity. The WHO [21] and SANS241 [22] recommends that EC values below 1500 µS/m and 1700 µS/m, respectively are suitable for drinking water, with DWAF [23] specifications recommending an EC below 400 µS/m being suitable for irrigation. 

All the boreholes from the two landfill sites had EC values that exceeded the DWAF specifications for irrigation over both seasons. All the boreholes in the northern landfill site had EC values exceeding the recommended limits by WHO [21] and SANS241 [22] over both seasons with borehole SB04 in the southern landfill site being the only borehole exceeding these limits. Similar to TDS concentrations, higher EC values were noted for autumn samples in comparison to the winter samples, and this can be attributed to the relationship that exists between these two parameters. According to Kumar et al. [25], elevated EC values are a result of high concentrations of ionic constituents that are present in the water. The two parameters are, therefore, used to describe the salinity levels of the water [26]. TOC and COD concentrations are an indication of the amount of organic matter in the water and are useful in indicating the degree of pollution [27]. Mean TOC and COD concentrations were much higher for borehole samples in the northern landfill site in comparison to those in the southern landfill site over both seasons. 

There are no given specifications for COD by both SANS241 [22] and WHO [21] for drinking water. However, SANS241 [22] recommends a TOC value below 10 mg/L for drinking water purposes. There are no given specifications for both COD and TOC values for irrigation purposes. According to Moore [27], there are no known effects of TOC on livestock and drinking water. Na, Ca, Mg, and Cl concentration for both seasons in the northern landfill groundwater samples are above the WHO standard. Arsenic and lead values were slightly above the SANS241 standard, probably due to the influence of leachate in the groundwater.

### 4.2. Microbiological Characteristics of Groundwater Samples from the Northern and Southern Landfill Sites

According to Tripathi and Sharma [28], coliforms and faecal coliforms are established indicator organisms that are reliable for the detection of faecal contamination in water, due to sewage disposal or other sources. The groundwater samples from the southern landfill site showed an elevated concentration of both total coliform and *E. coli* in comparison to the northern landfill site over both seasons. All the boreholes from the two landfill sites had total coliform concentrations exceeding the recommended limit of less than 10 mg/L for drinking water as stipulated by SANS241 [22] and WHO [21]. 

**Figure 9:**
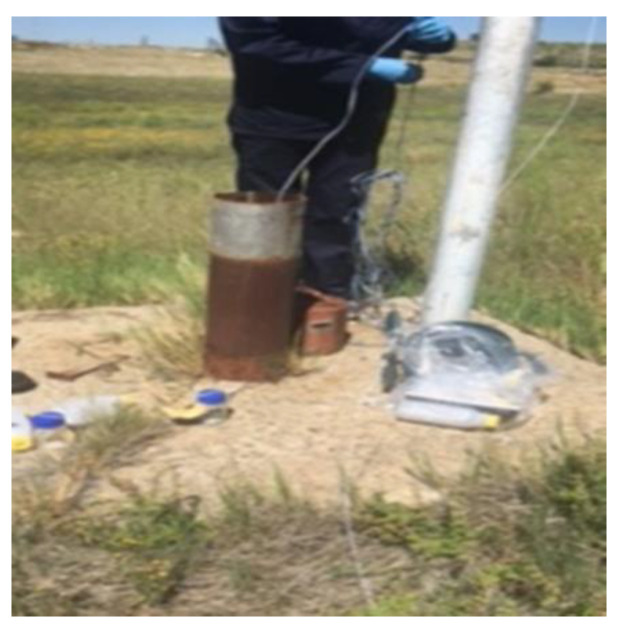
Well-constructed borehole in the northern landfill site.

**Figure 10:**
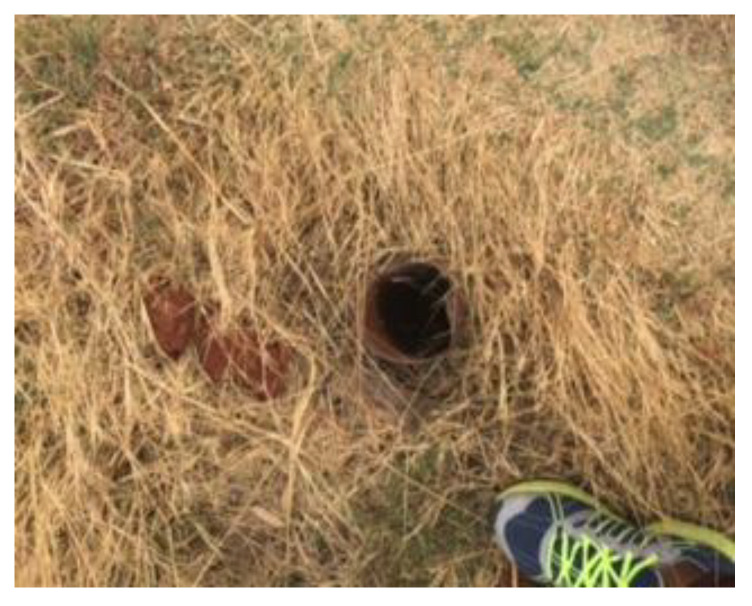
Poorly constructed borehole in the southern landfill site.

Elisante and Muzuka [29], stipulate that groundwater is prone to microbial contamination, due to human activities, such as soil fertility remediation, indiscriminate refuse and waste disposal, and the use of on-site sanitation facilities. All the boreholes in the northern landfill site had zero *E. coli* concentrations over both seasons and were, therefore, within the recommended drinking water limits as stipulated by SANS 241 [22] and WHO [21]. All the boreholes in the southern landfill site had notable *E. coli* concentrations, with borehole SB04 having the highest *E. coli* concentrations over both seasons. Findings by Elisante and Muzuka [29] concluded that differences in microbial contamination in boreholes can be attributed to the quality of borehole construction, as boreholes that were well constructed had low microbial contamination in comparison to those whose mouths were set at the ground surface. Figure 9 is a representation of the status of the boreholes in the northern landfill site with Figure 10 representing the borehole in the southern landfill site, whereby significant *E. coli* counts were noted. The poor state of the borehole in the southern landfill site might have resulted in the borehole being prone to microbial contamination.

### 4.3. The Impact of Geology (Rock Types and Hydrogeology) on the Percolation of Leachate into Groundwater

It is evident from the results and the discussion that the quality of the groundwater samples from the two landfill sites vary significantly, especially in terms of their physicochemical parameters and microbial quality. Although the two landfill sites both lack some required facilities of a modern landfill, are unlined, receive general “domestic” waste, located within the same geographical area and experience the same climatic conditions, the groundwater samples from the northern landfill site show more signs of contamination in comparison to the southern landfill site.

When dolerite intrusions intrude into the surrounding rocks, geological structures, such as joints, form along the contact zone with the country rocks [30]. Kale et al. [31], further illustrates that jointing and fracturing by way of interconnectivity also convey secondary porosity and permeability. The high permeability of the underlying dolerites, therefore, enables the percolation of contaminated leachate into the surrounding groundwater resources. According to Butler [12], the permeability of the rocks in the northern landfill site was measured by slug tests and was found to be fairly high with *k*-values of between 0.4 and 0.6 m/day. Butler [12] also further illustrates that the weathered dolerites underlying the landfill site are highly permeable, and this is the reason for the higher *k*-values as most of the boreholes were drilled through dolerite. With reference to the southern landfill site, slug tests established that the permeability of the underlying siltstones and shale is low with a *k*-value of 2.76 × 10^−3^ m/day [12]. A study of the northern landfill site that was conducted by the Institute of Groundwater Studies (UFS) concluded that the shallow water table, the high permeability of the underlying weathered dolerites, as well as the steep topography in the area was not suitable for siting a sanitary landfill [12]. A similar assessment was done for the southern landfill site, and the area was classified as a good location for landfill site because of the relatively deep water table, low permeability of the underlying geology and the gentle slope [12].

The comparison of the groundwater quality from the two landfill sites, as well as the underlying geology highlights the importance of a detailed investigation that must be done prior to selecting a site suitable for a landfill (see Supplementary Materials section for all the figures and tables). Although the two landfills are situated in similar geology, the presence of weathered dolerites beneath the northern landfill site resulted in the groundwater quality from the landfill site being of poorer quality in comparison to the southern landfill site.

## 5. Conclusions

This study assessed and compared the groundwater chemistry and quality from pollution monitoring boreholes in the vicinity of two landfill sites in Bloemfontein. A total of eight boreholes were sampled for water quality analysis. Three groundwater samples were taken from the southern landfill boreholes, while five boreholes samples were taken from the northern landfill boreholes. Hydrochemical facies analyses were determined using Piper and expanded Durov plots with Ca(Mg)SO_4_, Ca(Mg)HCO_3_ and Ca(Mg)Cl. The mixed hydrochemical facies point to constant changes of groundwater chemistry, due to the impact of the improperly designed landfills. 

The groundwater usability status was unsafe, due to higher concentrations of Ca, Mg, Cl, Na, Pb, As, TDS and EC which were above the SANS241 and WHO drinking water quality standard and DWAF specification for irrigation, an indication that the groundwater was unfit for drinking, domestic, and irrigation purposes, due to the impact of the landfills on the groundwater quality. The analysed parameters were higher in the boreholes samples of the northern landfill site and much lower in the boreholes samples of the southern landfill site. Boreholes in the northern landfill site are of poorer quality with reference to the general physicochemical parameters, while boreholes in the southern landfill site had a poorer quality based on microbiological parameters. 

Only one borehole in the southern landfill site had a high salinity hazard with the other two boreholes having a medium salinity hazard. There were no indications of significant metal concentrations in all sampled boreholes of both landfill sites. Comparison based on microbial parameters indicated that the boreholes in the northern landfill site had zero *E. coli* concentrations over both seasons, while boreholes in the southern landfill site had significant total coliform and *E. coli* concentrations over both seasons.

This study highlights that geology plays a significant role in the distribution of contaminants into groundwater systems. The presence of underlying dolerite in the northern landfill site resulted in the northern landfill site having a poorer water quality in comparison to the southern landfill site with respect to physicochemical parameters, while the poor state of the southern landfill boreholes made them more disposed to microbial contamination.

## Figures and Tables

**Table 1:** Water quality parameters groundwater samples collected in the autumn and winter seasons from the northern and southern landfill sites.

	Northern Landfill Site Autumn Season					Northern Landfill Site Winter Season					Southern Landfill Site Autumn			Southern Landfill Site Winter Season			Water Quality Guidelines		
Sampling site	NB03A	NB03B	NB07	NB06A	NB06B	NB03A	NB03B	NB07	NB06A	NB06B	SB04	SB08A	SB08B	SB04	SB08A	SB08B	SANS 2015	DWAF 1996	WHO (2015)
pH	7.6	7.8	7.4	7.3	7.5	7.8	7.7	7.4	7.4	7.4	7.5	8	7.9	7.0	7.7	7.6	5.0–9.7	6.5–8.4	6.5–8.5
TDS	1449.0	2435.0	4756.5	1537.0	1647.0	1364.5	2415.0	3432.0	1343.5	1417.0	1722.5	507.5	787.0	1480.0	597.0	641.5	1200.0	-	500.0
EC	1975.0	2950.0	6435.0	2305.0	2300.0	1930.0	2755.0	5570.0	2180.0	2165.0	2415.0	665.0	1105.0	2605.0	830.0	780.0	1700.0	<40.0	1500.0
COD	191.5	96.5	299.5	72.0	52.0	37.0	77.0	261.0	82.0	51.0	55.0	17.0	2.5	77.0	9.0	16.0	-	-	-
TOC	8.3	18.0	82.7	11.5	13.0	8.7	14.6	77.0	10.0	12.0	17.0	2.0	2.0	17.5	2.0	2.8	≤10.0	-	-
Ca	107.0	282.5	450.5	180.0	176.0	103.0	241.0	391.0	153.0	150.0	303.0	57.0	81.0	260.0	78.0	75.0	300.0	-	75.0
Mg	122.5	171.5	520.0	117.0	106.0	112.0	155.0	405.0	100.0	97.0	130.5	20.0	33.0	110.0	33.0	32.0	100.0	-	30.0
Na	168.0	264.0	351.5	169.0	219.5	116.0	235.0	272.0	128.0	165.0	78.0	77.0	86.0	63.0	62.0	59.0	≤200. 0	0.0–70.0	200.0
K	3.0	4.5	8.0	2.0	1.0	3.0	6.0	8.0	1.0	0.5	5.0	2.0	2.0	6.0	1.0	2.0	100.0	-	300.0
HCO_3_	174.0	362.5	939.5	699.5	708.5	98.0	478.0	647.0	596.0	569.0	621.0	237.0	276.0	469.0	259.0	334.0	-	-	-
SO_4_	535.0	998.0	31.0	19.0	5.0	536.0	975.0	34.0	11.0	8.0	29.0	27.0	54.0	1.0	51.0	48.o	≤500.0	-	500.0
Cl	300.0	318.5	2190.0	349.0	421.0	279.0	292.0	1656.0	347.0	412.0	540.0	58.0	123.0	567.0	80.0	75.0	≤300.0	0.0–105.0	250.0
Br	2.0	1.9	11.0	1.0	2.0	2.0	2.0	8.0	2.0	3.0	4.0	0.3	0.5	4.2	0.4	0.3	≤3.0	-	-
Mn	0.0	0.9	5.0	0.0	0.0	0.0	0.0	2.0	1.0	0.0	0.0	0.0	0.0	2.0	0.0	0.0	≤0.4	≤10.0	-
Cr	0.02	0.02	0.02	0.02	0.02	0.02	0.02	0.02	0.02	0.02	0.020	0.02	0.02	0.02	0.02	0.02	3.0	0.1	50.0
Cd	0.00	0.003	0.003	0.003	0.00	0.003	0.003	0.003	0.003	0.003	0.032	0.02	0.00	0.02	0.04	0.02	10.0	0.003	3.0
Co	0.02	0.02	0.02	0.02	0.02	0.02	0.02	0.02	0.02	0.02	0.02	0.02	0.02	0.02	0.04	0.02	50.0	0.05	-
Fe	0.05	0.05	0.04	0.02	0.14	0.6	0.09	0.19	0.02	0.14	0.06	0.02	0.02	0.04	0.04	0.02	300.0	5.0	-
Pb	0.015	0.015	0.03	0.02	0.014	0.015	0.015	0.02	0.022	0.014	0.015	0.02	0.01	0.02	0.04	0.05	0.01	0.2	-
Zn	0.02	0.02	0.02	0.02	0.02	0.02	0.02	0.02	0.02	0.02	0.02	0.02	0.02	0.02	0.02	0.02	0.5	1.0	-
As	0.02	0.02	0.02	0.02	0.02	0.02	0.02	0.17	0.02	0.02	0.02	0.02	0.02	0.02	0.02	0.02	0.01	0.1	10.0
V	0.078	0.3	2.3	0.02	0.09	0.02	0.008	2.5	1.02	0.09	2.4	0.03	0.01	1.7	0.03	0.06	4.0	0.1	-
Cu	0.028	0.03	0.04	0.02	0.16	0.02	0.029	0.04	0.02	0.01	0.018	0.01	0.01	0.03	0.01	0.02	2.0	0.2	-
se	0.02	0.02	0.02	0.02	0.02	0.02	0.02	0.02	0.02	0.02	0.02	0.02	0.02	0.02	0.02	0.02	0.010	0.02	40.0
Total coliform	98.5	2420.0	2420.0	23.0	517.0	2420.0	2420.0	3.0	38.5	36.0	242.0	1120.0	43.0	242.0	1300.0	411.0	≤10.0	varies	10.0
*Escherichia coli*	0.0	0.0	0.0	0.0	0.0	0.0	0.0	0.0	0.0	0.0	307.0	1.0	1.0	6.5	71.5	7. 0	0.0	0.0	0.0

Mean values for TDS, TOC and COD are expressed in mg/L, EC in mS/m, and pH in pH units. Total coliform and *E. coli* expressed in cfu/100 mL. Water guidelines as per SANS 241:2015 drinking water standards, World Health Organisation (WHO, 2011) and DWAF specifications for irrigation.

**Table 2:** Comparison of the average physicochemical and microbiological parameters of the two landfill sites over two seasons.

	Northern Landfill Site		Southern Landfill Site	
Parameter	Autumn	Winter	Autumn	Winter
pH	7.5	7.5	7.8	7.4
TDS	2365.0	1994.7	1005.0	906.0
EC	3160.0	2920.0	1400.0	1400.0
TOC	27.0	24.0	6.9.0	7.5
COD	142.0	101.0	24.0	34.0
Total coliform	1095.0	928.0	1194.5	1377.0
*E. coli*	0.0	0.0	103.0	28.0

Mean values for total coliform and *E. coli* given in cfu/100 mL.

## Supplementary Materials

The following are available online. Figure S1: Map illustrating the two landfill sites in Bloemfontein and the location of the pollution monitoring boreholes in the vicinity of the two landfill sites, Figure S2: Schematic presentation of the Karoo Supergroup sequence, Figure S3: Piper diagram for the groundwater samples in the northern landfill site for the two seasons, Figure S4: Piper diagram for the groundwater samples in the southern landfill site for the two seasons. Figure S5: Expanded Durov diagram for groundwater samples in the northern landfill site for the two seasons, Figure S6: Expanded Durov diagram for groundwater samples in the southern landfill site for the two seasons, Figure S7: Salinity hazard (SAR) diagram for the boreholes in the northern landfill site, Figure S8: SAR diagram for the boreholes in the southern landfill site, Figure S9: Well-constructed borehole in the northern landfill site, Figure S10: Poorly constructed borehole in the southern landfill site. Table S1: Water quality parameters groundwater samples collected in the autumn and winter seasons from the northern and southern landfill sites, Table S2: Comparison of the average physicochemical and microbiological parameters of the two landfill sites over two seasons.
