# Supplementary material for: The Influence of Non-Engineered Municipal Landfills on Groundwater Chemistry and Quality in Bloemfontein, South Africa
Source: Molecules. 2020 Nov 28;25(23):5599. doi: 10.3390/molecules25235599 (PMC7731076; doi:10.3390/molecules25235599)
Supplement: Supplementary file 1 [file molecules-25-05599-s001.zip › mdpi_references_guide_v5.pdf]

Reference List and Citations
Style Guide for MDPI Journals

MDPI's style for citations and references lists are widely based on the style used by the American Chemical Society. Please refer to the ACS Style Guide [1], or enquire with a journal's editorial office, if you have any questions regarding preparation of citations and references lists.

Citations

Reference citation numbers should be placed in square brackets, i.e. [ ], and placed inside the punctuation, for example [4]. or [1–3], and all the references should be listed separately and as the last section at the end of the manuscript. Do not use superscript text to indicate citations.

References List

General Considerations

To allow automated treatment of bibliographic information, references and citations must be prepared according to the following general rules.

- Avoid using nested references:

  > 1. (a) reference 1; (b) reference 2; (c) reference 3.

  Instead use single references:

  > 1. reference 1.
  > 2. reference 2.
  > 3. reference 3.

  And cite as in the text:

  > … [1–3].

- Avoid including notes with the references, e.g. do not use:

  > 10. Díaz et al. recently reported a multigram display of azide and cyanide components on a versatile scaffold, see: Díaz, D.D.; Converso, A.; Sharpless, K.B.; Finn, M.G. 2,6-Dichloro-9-thiabicyclo[3.3.1]nonane: Multigram Display of Azide and Cyanide Components on a Versatile Scaffold. *Molecules* **2006**, *11*, 212–218.

  Instead use the reference:

  > 10. Díaz, D.D.; Converso, A.; Sharpless, K.B.; Finn, M.G. 2,6-Dichloro-9-thiabicyclo[3.3.1]nonane: Multigram Display of Azide and Cyanide Components on a Versatile Scaffold. *Molecules* **2006**, *11*, 212–218, doi:10.3390/11040212.

  And add the note to the text together with the citation:

  > … recently reported by Díaz et al. [10].

Rules Applying to All Types of References

Author Information

- Authors must be listed in the same order as they appear in the original document. Different authors are separated with semicolons (';'):

> Author 1; Author 2; Author 3; etc.

- For every author, list the last name first, then the first letter of the first name and, if available, the first letter of the middle name.
- Last names should be separated from first and middle name by a comma, first and middle names should end with a period:

> Lastname, F.M.; Lastname, F.M.; etc.

- Special cases "van", "von", "de" and others:

> von Lastname, F.M.; van Lastname, F.M.; etc.

- Author names with suffixes "Jr.", "Sr.", "II", etc.:

> Lastname, F.M., Jr.; Lastname, F.M., Sr.; etc.

- For documents co-authored by a large number of persons (more than 10 authors), you can either cite all authors, or cite the first ten authors, then add a semicolon and add 'et al.' at the end:

> Author 1; Author 2; Author 3; Author 4; Author 5; Author 6; Author 7; Author 8; Author 9; Author 10; et al.

Editor Information

- List editors' names in the same way as authors' names, except that multiple editors are separated by commas instead of semicolons:

> Editor 1, Editor 2, Editor 3, etc.

- Add 'Ed.' (one editor) or 'Eds.' (multiple editors) at the end of the list:

> Editor 1, Ed.

or

> Editor 1, Editor 2, Editor 3, Eds.

Common Types of References

- Journal/Periodical Articles
- Books
  - with editors
  - without editors
- Conferences or Meetings
  - Proceedings
  - Oral presentations without published material
- Websites/online resources

For more reference types refer to the ACS Style Guide [1].

Journal/Periodical Articles

Format:

> Author 1; Author 2; Author 3; etc. Title of the article. *Journal Abbreviation* **Year**, *Volume*, Firstpage–Lastpage, doi:prefix/suffix.

Example:

> 15. Díaz, D.D.; Converso, A.; Sharpless, K.B.; Finn, M.G. 2,6-Dichloro-9-thiabicyclo[3.3.1]nonane: Multigram Display of Azide and Cyanide Components on a Versatile Scaffold. *Molecules* **2006**, *11*, 212–218, doi:10.3390/11040212.

Journal abbreviation field:

- Use ISO4 abbreviated titles for journals. A list of abbreviations is available from:
  - PubMed/MEDLINE, see ftp://ftp.ncbi.nih.gov/pubmed/J_Medline.txt
  - MDPI journals that use one word titles such as *Sensors*, *Molecules*, *Entropy*, *Energies*, etc. are not abbreviated.
  - Abbreviations for other MDPI journals can be found from the latest articles on the journal homepage, e.g. http://www.mdpi.com/journal/ijms.

Pagination field:

- Use inclusive pagination, with starting and ending page numbers
- For single page articles (abstracts), only add one number
- Some online-only Publishers (e.g. Biomed Central) recently started to use article numbers instead of page numbers. For such articles without page numbers, reference the article number and the number of pages in the PDF version:

> ArticleNumber:1–ArticleNumber:NumberOfPages

E.g., for an article numbered '43' and containing 13 pages in the PDF version, use:

> 43:1–43:13

Avoid citing only the article number, as this reference will look like a one-page abstract.

Books

Books without editors

Format:

> Author 1; Author 2; Author 3; etc. *Title of the Book*, Edition (if available); Publisher: City, Country, Year; Chapter (optional), pp. Page range (optional), ISBN XXX-XXX-XXX-XXX-X.

Example:

> Desiraju, G.R.; Steiner, T. *The Weak Hydrogen Bond in Structural Chemistry and Biology*, 2nd ed.; Oxford University Press: New York, NY, USA, 1999; pp. 10–25, ISBN 978-01-9850-970-7.

Title and edition field:

- Unlike journal article titles, book titles are in *italic*.
- The edition number is appended at the end of the title, separated with by a comma, with the edition number as an ordinal number (1st, 2nd, 3rd, 4th, etc.) followed by 'ed.'. The letters in the ordinal number should <u>not</u> be put in superscript. 'ed.' should <u>not</u> be capitalized to avoid confusion with the abbreviation for editors (that is 'Ed.' or 'Eds.').

Year:

- For books, year information is not in boldface

Pagination:

- Use pp. to refer to a page range or multiple pages, e.g. pp. 25–67 or pp. 5, 110–123, 185
- Use p. to refer to one single page
- Omit pagination information if the whole book is the reference

Place of Publication:

- Use the Publisher's headquarters' city name or, if the book is published by a subsidiary, use the subsidiary's city name
- For cities in the USA, add the city name, the abbreviation of the state, and 'USA':
  Springfield, IL, USA
- For all other countries, add the city name, and the country name:
  London, UK

Books with editors

Format:

> Author 1; Author 2; Author 3; etc. Title of the contribution. In *Title of the Book*, Edition (if available); Editor 1, Editor 2, Eds.; Publisher: City, Country, Year; Volume (optional), pp. Pages (optional), ISBN XXX-XXX-XXX-XXX-X.

Example:

> Dodd, J.S.; Solla, L.; Bérard, P.M. References. In *The ACS Style Guide*, 3rd ed.; Coghill, A., Garson, L., Eds.; American Chemical Society: Washington, DC, USA, 2006; Volume 1, pp. 292–325, ISBN 978-0-8412-3999-9.

Title and Edition Field:

- Unlike journal article titles, book titles are in *italic*.
- The edition number is appended at the end of the title, separated with by a comma, with the edition number as an ordinal number (1st, 2nd, 3rd, 4th, etc.) followed by ‘ed.’. The letters in the ordinal number should <u>not</u> be put in superscript. ‘ed.’ should <u>not</u> be capitalized to avoid confusion with the abbreviation for editors (that is ‘Ed.’ or ‘Eds.’).

Editor Field:

- Note that multiple editors are separated by commas (unlike authors, which are separated by semicolons).

Year:

- For books, year information is not in boldface.

Pagination:

- Use pp. to refer to a page range or multiple pages, e.g. pp. 25–67 or pp. 5, 110–123, 185.
- Use p. to refer to one single page.
- Omit pagination information if the whole book is the reference.

Place of Publication:

- Use the Publisher’s headquarters’ city name or, if the book is published by a subsidiary, use the subsidiary’s city name
- For cities in the USA, add the city name, the abbreviation of the state, and ‘USA’:

  Springfield, IL, USA
- For all other countries, add the city name, and the country name:

  London, UK

Conferences or Meetings

Proceedings

Format:

> Author 1; Author 2; Author 3; etc. Title of Presentation. In Proceedings of the Name of the Conference, Location of Conference, Country, Date of Conference (Day Month Year); Abstract Number (optional), Pagination (optional).

Example:

> Chum, O.; Philbin, J.; Zisserman, A. Near duplicate image detection: Min-Hash and tf-idf weighting. In Proceedings of the 19th British Machine Vision Conference (BMVC 2008), Leeds, UK, 1–4 September 2008; pp. 812–815.

Format:

> Author 1; Author 2; Author 3; etc. Title of Presentation. In *Title of the Collected Work, Proceedings of the Name of the Conference, Location of Conference, Country, Date of Conference (Day Month Year)*; Editor 1, Editor 2, Eds. (if available); Publisher: City, Country, Year; Abstract Number (optional), Pagination (optional).

Example:

> Beebe, N. Digital forensic research: The good, the bad and the unaddressed. In *Advances in Digital Forensics V, Proceedings of the Fifth IFIP WG 11.9 International Conference on Digital Forensics, Orlando, FL, USA, 26–28 January 2009*; Springer: Berlin/Heidelberg, Germany, 2009; Volume V, pp. 17–36.

Oral presentations without published material

Format:

> Author 1; Author 2; Author 3; etc. Title of Presentation (if any). Presented at the Name of Conference, Location of Conference, Country, Date of Conference; Paper number (if available).

Example:

> Zhang, Z.; Chen, H.; Zhong, J.; Chen, Y.; Lu, Y. ZnO Nanotip-based QCM Biosensors. Presented at the IEEE International Frequency Control Symposium and Exposition, Miami, FL, USA, June 2006.

Website and Online Resources

Format:

> Author (if available). Title of the webpage (if available). Available online: http://... (accessed on date).

Example:

> International Union of Pure and Applied Chemistry Home Page. Available online: http://www.iupac.org/dhtml_home.html (accessed on 24 April 2005).

References

1. Dodd, J.S.; Solla, L.; Bérard, P.M. References. In *The ACS Style Guide*, 3rd ed.; Coghill, A., Garson, L., Eds.; American Chemical Society: Washington, DC, USA, 2006; pp. 292–325, ISBN 978-0-8412-3999-9.

Version 5, 4 September 2018
