# Supplementary material for: The Influence of Non-Engineered Municipal Landfills on Groundwater Chemistry and Quality in Bloemfontein, South Africa
Source: Molecules. 2020 Nov 28;25(23):5599. doi: 10.3390/molecules25235599 (PMC7731076; doi:10.3390/molecules25235599)
Supplement: Supplementary file 1 [file molecules-25-05599-s001.zip › winter north and south.pdf]

Institute for Groundwater Studies

University of the Free State

IGS Laboratory Services, Dekaan Street (Campus)

339, BLOEMFONTEIN, 9300

+27-(0)51 - 401 2317

+27-(0)51 - 401 3005

E-mail: igslab@ufs.ac.za

sanas testing laboratory T0695

Test Report

Case no: 2018 - 546

19/06/2018

Determinand	Units	Methods used	South African National Standard (SANS) 241:2006&2015 for drinking water (partial)	Client sample name:																	
				NB 03 A 19/06/18	NB 03 A 19/06/18	NB 03 B 19/06/18	NB 03 B 19/06/18	NB 06 A 19/06/18	NB 06 A 19/06/18	NB 06 B 19/06/18	NB 06 B 19/06/18	NB 07 19/06/18	NB 07 19/06/18	SB 07 A 19/06/18	SB 07 A 19/06/18	SB 08 A 19/06/18	SB 08 A 19/06/18	SB 08 B 19/06/18	SB 08 B 19/06/18	Surface North 19/06/18	Surface North 19/06/18
			Class 1 (Recommended levels) Class 2 (Maximum allowable for limited time) ** EU standard	Lab number:																	
				546-1 (a)	546-1 (b)	546-2 (a)	546-2 (b)	546-3 (a)	546-3 (b)	546-4 (a)	546-4 (b)	546-5 (a)	546-5 (b)	546-6 (a)	546-6 (b)	546-7 (a)	546-7 (b)	546-8 (a)	546-8 (b)	546-9 (a)	546-9 (b)
			Value	Value	Value	Value	Value	Value	Value	Value	Value	Value	Value	Value	Value	Value	Value	Value	Value	Value	Value
**Chemical report**																					
pH #	pH units	Chem-TM06	5.5 tot 9.7	7.89	7.88	7.79	7.72	7.47	7.39	7.35	7.43	7.25	7.17	7.16	7.13	7.71	7.63	7.65	7.61	7.93	8.00
Electrical conductivity #	mS/m	Chem-TM06	≤ 170	194	181	275	283	218	216	217	214	557	543	261	257	83.98	82.84	76.93	78.29	38.8	38.7
Calcium as Ca #	mg/L	Chem-TM02	≤150 - 300	119	87	236	246	155	151	160	141	385	396	257	262	75.0	80.4	76.0	74.2	41.2	42.3
Magnesium as Mg #	mg/L	Chem-TM02	≤70 - 100	115	108	154	156	101	98	103	91	403	407	110	110	32.1	34.0	32.8	32.6	15.8	16.3
Sodium as Na #	mg/L	Chem-TM02	≤ 200	127	105	222	247	129	127	170	159	270	274	63	62	57.8	59.9	62.1	61.4	17.1	17.7
Potassium as K #	mg/L	Chem-TM02	≤50 - 100	3.3	3.0	5.7	4.6	1.0	0.9	0.5	0.6	7.9	7.8	4.7	4.5	1.4	1.4	1.8	1.8	4.7	5.0
P-Alkalinity #	mg/L	Chem-TM06		0	0	0	0	0	0	0	0	0	0	0	0	0	0	0	0	0	0
M-Alkalinity #	mg/L	Chem-TM06		129	73	464	492	601	591	564	573	652	669	474	465	254	264	331	338	128	129
Fluoride as F #	mg/L	Chem-TM01	≤ 1.5	<0.1	<0.1	<0.1	0.17	<0.1	<0.1	<0.1	<0.1	<0.1	<0.1	<0.1	<0.1	0.65	0.50	0.37	0.42	0.04	0.07
Chloride as Cl #	mg/L	Chem-TM01	≤ 300	288	269	290	293	347	346	408	415	1677	1636	568	565	79.8	79.7	75.4	74.1	22.6	22.5
Nitrite as N #	mg/L	Chem-TM01		-0.1	-0.1	-0.1	-0.1	-0.1	-0.1	-0.1	-0.1	-0.1	-0.1	-0.1	-0.1	-0.01	-0.01	-0.01	-0.01	-0.01	-0.01
Bromide as Br #	mg/L	Chem-TM01	**≤3	2.37	2.71	1.58	2.05	1.80	1.61	2.51	2.55	8.24	8.54	4.24	4.20	0.41	0.39	0.31	0.31	0.03	0.10
Nitrate as N #	mg/L	Chem-TM01	≤ 11	10.49	3.40	3.40	5.19	0.02	0.14	0.05	0.06	0.04	0.11	0.01	0.07	5.76	5.86	2.40	2.93	0.05	0.06
Phosphate as PO_4 #	mg/L	Chem-TM01	*≤15.33	-1	-1	-1	-1	-1	-1	1.56	-1	-1	-1	-1	1.05	-0.1	-0.1	0.12	-0.1	-0.1	-0.1
Sulphate as SO_4 #	mg/L	Chem-TM01	≤ 500	537.3	500.7	975.0	953.2	9.7	12.3	8.6	8.2	30.7	34.9	1.2	1.3	51.2	51.4	48.9	46.5	47.6	47.6
Calcium Hardness #	mg/L	calculated	≤375 - 750	297	217	590	616	389	377	400	352	962	990	643	654	187	201	190	185	103	106
Magnesium Hardness #	mg/L	calculated	≤287 - 410	472	442	632	639	412	401	423	372	1652	1669	450	452	132	139	134	134	65	67
Total Hardness as $CaCO_3$ #	mg/L	calculated	≤662 - 1160	768	659	1223	1255	801	778	823	724	2615	2659	1093	1106	319	340	324	319	168	173
Total Dissolved Solids #	mg/L	calculated	≤ 1200	1365	1161	2362	2415	1344	1327	1418	1388	3433	3432	1480	1475	578	598	639	642	277	281
Total organic carbon as C #	mg/L	Chem-TM24	≤ 10	8.76	7.82	14.68	14.49	10.26	10.59	12.26	13.15	77.4	77.1	17.51	16.78	2.29	2.23	2.85	2.32	9.25	8.27
Chemical oxygen demand #	mg/L			38	29	77	69	82	83	50	64	256	262	71	78	9	9	16	11	30	32
Aluminium as Al #	mg/L	Chem-TM02	≤ 0.300	<0.020	<0.020	<0.020	<0.020	<0.020	<0.020	<0.020	<0.020	<0.020	<0.020	<0.020	<0.020	<0.020	<0.020	<0.020	<0.020	0.071	0.083
Arsenic as As #	mg/L	Chem-TM02	≤ 0.010	<0.020	<0.020	<0.020	<0.020	<0.020	<0.020	<0.020	<0.020	<0.020	<0.020	0.024	0.025	<0.020	<0.020	<0.020	<0.020	<0.020	<0.020
Barium as Ba #	mg/L	Chem-TM02	≤ 0.700	0.010	0.007	0.003	0.003	0.042	0.042	0.029	0.028	0.017	0.017	0.181	0.186	0.132	0.139	0.098	0.087	0.038	0.039
Boron as B #	mg/L	Chem-TM02	≤ 2.400	0.068	0.023	0.144	0.162	<0.040	<0.040	<0.040	<0.040	0.107	0.080	<0.040	<0.040	0.046	0.050	<0.040	<0.040	<0.040	<0.040
Cadmium as Cd #	mg/L	Chem-TM02	≤ 0.003	0.007	0.006	<0.003	<0.003	<0.003	<0.003	<0.003	<0.003	<0.003	<0.003	<0.003	<0.003	<0.003	<0.003	<0.003	<0.003	<0.003	<0.003
Cobalt as Co #	mg/L	Chem-TM02	≤ 0.500	<0.020	<0.020	<0.020	<0.020	<0.020	<0.020	<0.020	<0.020	<0.020	<0.020	<0.020	<0.020	<0.020	<0.020	<0.020	<0.020	<0.020	<0.020
Chromium as Cr #	mg/L	Chem-TM02	≤ 0.050	<0.020	<0.020	<0.020	<0.020	<0.020	<0.020	<0.020	<0.020	<0.020	<0.020	<0.020	<0.020	<0.020	<0.020	<0.020	<0.020	<0.020	<0.020
Copper as Cu #	mg/L	Chem-TM02	≤ 2.000	0.028	0.032	0.033	0.035	0.022	0.021	0.016	0.025	0.040	0.040	0.038	0.027	0.011	0.003	0.000	0.001	0.010	0.015
Iron as Fe #	mg/L	Chem-TM02	≤ 2.000 (chronic health)	0.053	0.033	0.057	0.067	0.029	0.023	0.140	0.221	0.047	0.064	0.046	0.034	0.043	<0.020	0.024	0.025	0.108	0.122
	mg/L	Chem-TM02	≤ 0.300 (aesthetic)																		
Manganese as Mn #	mg/L	Chem-TM02	≤ 0.400 (Chronic health)	0.087	0.185	0.368	0.257	1.044	0.966	0.096	0.085	2.643	2.399	1.609	1.724	<0.020	<0.020	<0.020	<0.020	0.022	0.022
	mg/L	Chem-TM02	≤ 0.100 (Aesthetic)																		
Nickel as Ni #	mg/L	Chem-TM02	≤ 0.070	0.061	0.028	0.113	0.096	0.127	0.120	0.074	0.070	0.273	0.231	0.089	0.081	0.064	0.058	0.053	0.043	0.023	<0.020
Lead as Pb #	mg/L	Chem-TM02	≤ 0.010	<0.015	<0.015	<0.015	<0.015	0.022	<0.015	0.014	0.032	0.033	0.035	0.020	0.027	0.048	0.021	0.042	0.018	0.018	<0.015
Selenium as Se #	mg/L	Chem-TM02	≤ 0.040	<0.020	<0.020	<0.020	<0.020	<0.020	<0.020	<0.020	<0.020	<0.020	<0.020	<0.020	<0.020	<0.020	<0.020	<0.020	<0.020	<0.020	<0.020
Stronsium as Sr #	mg/L	Chem-TM02		0.398	0.255	0.867	0.915	0.461	0.449	0.410	0.381	1.125	1.040	2.121	2.127	0.644	0.681	0.545	0.507	0.104	0.106
Silicon as Si #	mg/L	Chem-TM02		4.25	2.34	12.67	13.08	19.45	18.76	15.01	13.37	15.94	14.64	13.34	13.22	14.22	15.88	11.91	12.33	5.33	5.31
Vanadium as V #	mg/L	Chem-TM02	≤ 0.200	0.078	0.170	0.353	0.246	1.027	0.944	0.090	0.077	2.551	2.313	1.669	1.770	0.026	0.038	0.036	0.032	0.049	0.050
Zinc as Zn #	mg/L	Chem-TM02	≤ 5.000	<0.020	<0.020	<0.020	<0.020	<0.020	<0.020	<0.020	<0.020	<0.020	<0.020	0.020	0.024	<0.020	<0.020	<0.020	<0.020	<0.020	<0.020
**Bacterial report:**																					
Total coliform	cfu/100ml	BAC-TM02	≤10	1300	>2420	2420	>2420	39	33	31	26	0	3	2420	>2420	1300	488	411	270	687	1553
E. coli	cfu/100ml	BAC-TM02	0	0	0	0	0	0	0	0	0	0	0	6	7	71	51	5	7	13	18

Note:

Results marked with (#) in this report, are not included in the SANAS Schedule of Accreditation for this laboratory.

Parameters higher than the specifications, are clearly **marked**. Table with specifications included for comparison.

Bacteriological results obtained from samples older than the prescribed 24 hours may be inaccurate.

Signature: ___________________________

Dr L Deysel (Technical signatory / Technical manager)

END OF REPORT

From: DWAF Domestic use guidelines			
**Description of Hardness**	**Hardness Range**	**Description of Hardness**	**Hardness Range**
Soft	0 - 50	Moderately harc	150 - 200
Moderately soft	50 - 100	Hard	200 - 300
Slightly hard	100 - 150	Very hard	> 300
