# Supplementary material for: The Influence of Non-Engineered Municipal Landfills on Groundwater Chemistry and Quality in Bloemfontein, South Africa
Source: Molecules. 2020 Nov 28;25(23):5599. doi: 10.3390/molecules25235599 (PMC7731076; doi:10.3390/molecules25235599)
Supplement: Supplementary file 1 [file molecules-25-05599-s001.zip › autumn south.pdf]

Institute for Groundwater Studies

University of the Free State

IGS Laboratory Services, Dekaan Street (Campus)

339, BLOEMFONTEIN, 9300

+27-(0)51 - 401 2317

: +27-(0)51 - 401 3005

E-mail: igslab@ufs.ac.za

Sec 5.10 F1

Revision 9

sanas Testing Laboratory T0695

Test Report

Case no: 2018 - 210 02/03/2018

Determinand	Units	Methods used	South African National Standard (SANS) 241:2006&2015 for drinking water (partial) Class 1 (Recommended levels) Class 2 (Maximum allowable for limited time) ** EU standard	Livestock Watering Recommended values	Dwaf specifications for irrigation	SB 08 A (1) 02-03-18	SB 08 A (2) 02-03-18	SB 05 A 02-03-18	SB 05 B 02-03-18	SB 08 B (1) 02-03-18	SB 08 B (2) 02-03-18	NB 07
					Lab number:	210-10	210-11	210-12	210-13	210-14	210-15	210-16
			Value	Value	Value	Value	Value	Value	Value	Value	Value	Value
**Chemical report**												
COD #	mg/L	Chem-TM04				17	15	55	50	2	3	299
Total organic carbon as C #	mg/L	Chem-TM24	≤ 10			2.42	2.34	15.9	17.0	2.19	2.36	81.5
Ammonia as N #	mg/L	Chem-TM18	≤ 1.5			<0.5	<0.5	<0.5	<0.5	<0.5	<0.5	0.63
pH #	pH units	Chem-TM06	5.5 tot 9.7		6.5 - 8.4	8.14	8.01	7.54	7.34	7.98	8.09	7.45
Electrical conductivity #	mS/m	Chem-TM06	≤ 170		<40	67.6	66.9	238	241	78.7	110	643
Calcium as Ca #	mg/L	Chem-TM02	≤150 - 300	≤1000		56.5	57.0	298	308	68.9	92.5	451
Magnesium as Mg #	mg/L	Chem-TM02	≤70 - 100	≤500		20.0	20.3	129	132	31.3	35.2	520
Sodium as Na #	mg/L	Chem-TM02	≤ 200	≤2000	0 - 70	79.0	74.2	77.2	78.6	65.7	107	352
Potassium as K #	mg/L	Chem-TM02	≤50 - 100			1.57	1.42	5.16	5.29	2.00	2.29	8.22
P-Alkalinity #	mg/L	Chem-TM06				0	0	0	0	0	0	0
M-Alkalinity #	mg/L	Chem-TM06				238	236	617	624	254	298	1216
Fluoride as F #	mg/L	Chem-TM01	≤ 1.5	≤2		0.93	0.98	0.25	0.07	0.55	0.04	<0.20
Chloride as Cl #	mg/L	Chem-TM01	≤ 300	≤1500	0 - 105	59	57	537	542	85	161	2176
Nitrite as N #	mg/L	Chem-TM01				<0.01	<0.01	<0.1	<0.1	<0.01	<0.1	<0.2
Bromide as Br #	mg/L	Chem-TM01	**≤3			0.30	0.30	3.86	4.23	0.40	0.53	11.18
Nitrate as N #	mg/L	Chem-TM01	≤ 11	≤22.5	0 - 5	0.13	0.09	0.76	0.63	1.99	7.92	<1
Phosphate as PO_4 #	mg/L	Chem-TM01	*≤15.33			<0.1	<0.1	<1	<1	<0.1	<1	<2
Sulphate as SO_4 #	mg/L	Chem-TM01	≤ 500	≤1000		52.8	52.8	29.8	27.8	52.2	56.4	28.6
Calcium Hardness #	mg/L	calculated	≤375 - 750			141	142	744	769	172	231	1128
Magnesium Hardness #	mg/L	calculated	≤287 - 410			82	83	527	543	128	144	2133
Total Hardness as $CaCO_3$ #	mg/L	calculated	≤662 - 1160			223	226	1271	1312	300	376	3261
Total Dissolved Solids #	mg/L	calculated	≤ 1200			508	499	1697	1722	568	786	4757
Sodium Adsorption Ratio(SAR) #		calculated			0 - 1.5	2.30	2.15	0.94	0.94	1.65	2.40	2.68
Aluminium as Al #	mg/L	Chem-TM02	≤ 0.300	≤5	0 - 0.5	<0.020	<0.020	<0.020	<0.020	<0.020	<0.020	<0.020
Arsenic as As #	mg/L	Chem-TM02	≤ 0.010	≤1	0 - 0.1	<0.020	<0.020	<0.020	<0.020	<0.020	<0.020	<0.020
Barium as Ba #	mg/L	Chem-TM02	≤ 0.700			0.120	0.120	0.177	0.183	0.079	0.145	<0.020
Boron as B #	mg/L	Chem-TM02	≤ 2.400		0 - 0.5	0.209	0.204	0.081	0.072	0.094	0.116	0.181
Cadmium as Cd #	mg/L	Chem-TM02	≤ 0.003			<0.003	<0.003	<0.003	<0.003	<0.003	<0.003	<0.003
Cobalt as Co #	mg/L	Chem-TM02	≤ 0.500			<0.020	<0.020	<0.020	<0.020	<0.020	<0.020	<0.020
Chromium as Cr #	mg/L	Chem-TM02	≤ 0.050	≤0.05		<0.020	<0.020	<0.020	<0.020	<0.020	<0.020	<0.020
Copper as Cu #	mg/L	Chem-TM02	≤ 2.000	≤0.5	0 - 0.2	0.011	0.008	0.018	0.018	0.013	0.015	0.042
Iron as Fe #	mg/L	Chem-TM02	≤ 2.000 (chronic health)	≤10	0 - 0.5	<0.020	<0.020	0.061	0.041	<0.020	<0.020	0.090
	mg/L	Chem-TM02	≤ 0.300 (aesthetic)									
Manganese as Mn #	mg/L	Chem-TM02	≤ 0.400 (Chronic health)			0.034	0.033	2.415	2.547	<0.020	0.070	5.366
	mg/L	Chem-TM02	≤ 0.100 (Aesthetic)	≤10								
Nickel as Ni #	mg/L	Chem-TM02	≤ 0.070			<0.020	<0.020	0.026	0.028	<0.020	<0.020	0.223
Molybdenum as Mo #	mg/L	Chem-TM02		≤0.01	0 - 0.02	<0.020	<0.020	<0.020	<0.020	<0.020	<0.020	<0.020
Lead as Pb #	mg/L	Chem-TM02	≤ 0.010	≤0.1	0 - 0.2	<0.015	<0.015	<0.015	<0.015	<0.015	<0.015	<0.015
Selenium as Se #	mg/L	Chem-TM02	≤ 0.040			<0.020	<0.020	<0.020	<0.020	<0.020	<0.020	<0.020
Vanadium as V #	mg/L	Chem-TM02	≤ 0.200			0.032	0.031	2.336	2.491	<0.010	0.068	5.178
Zinc as Zn #	mg/L	Chem-TM02	≤ 5.000	≤20	0 - 1.0	<0.020	<0.020	<0.020	<0.020	<0.020	<0.020	<0.020
**Bacterial report:**												
Total coliform	cfu/100ml	BAC-TM02	≤10	≤10	specifications will vary with the kind of crop irrigated.	649	1120	>2420	>2420	43	36	
E. coli	cfu/100ml	BAC-TM02	0	0		1	1	308	261	1	0	

Note:

Results marked with (#) in this report, are not included in the SANAS Schedule of Accreditation for this laboratory.

Parameters higher than the specifications, are clearly marked. Tables with specifications included for comparison.

Bacteriological results obtained from samples older than the prescribed 24 hours may be inaccurate.

Signature: ___________________________

Dr L Deysel (Technical signatory / Technical manager)

END OF REPORT

From: DWAF Domestic use guidelines

Description of Hardness	Hardness Rang	Description of Hardness	Hardness Range
Soft	0 - 50	Moderately hard	150 - 200
Moderately soft	50 - 100	Hard	200 - 300
Slightly hard	100 - 150	Very hard	> 300
Moderately hard	150 - 200		
Hard	200 - 300		
Very hard	> 300		

C_2: Medium salt content. Provision must be made for leaching of salts and plants sensitive to brakishness must be avoided.
must be avoided.
S_1: Low Sodium: Can be used for irrigation - contains a low brakish danger.

Irrigation water - type

EC mS/m

**0-25**	C_1: Low salt content: No danger for brakishness on well drained soils.
**26-75**	C_2: Medium salt content. Provision must be made for leaching of salts and plants sensitive to brakishness must be avoided.
**76-225**	C3: High salt content. Can only be used on soils with a good drainage. Leaching is needed periodically and plants sensitive to brakish water must be avoided.
**>225**	C4: Very high salt content. Not fit for irrigation under normal conditions. Can be used in an emergency on sandy soils.

SAR

**0 - 10**	S_1: Low Sodium: Can be used for irrigation - contains a low brakish danger.
**10 - 18**	S2: Medium Sodium: Mainly to be used on sandy soils with a very good drainage.
**18 - 26**	S3: High Sodium: Not to be used on soils with limited drainage.
**26 - 34**	S_4: Very high sodium: Not fit for irrigation because of the high sodium content.
