# Supplementary material for: The Influence of Non-Engineered Municipal Landfills on Groundwater Chemistry and Quality in Bloemfontein, South Africa
Source: Molecules. 2020 Nov 28;25(23):5599. doi: 10.3390/molecules25235599 (PMC7731076; doi:10.3390/molecules25235599)
Supplement: Supplementary file 1 [file molecules-25-05599-s001.zip › autumn north.pdf]

Institute for Groundwater Studies

University of the Free State

IGS Laboratory Services, Dekaan Street (Campus)

✉ 339, BLOEMFONTEIN, 9300

☎ +27-(0)51 - 401 2317

📠: +27-(0)51 - 401 3005

E-mail: igslab@ufs.ac.za

sanas Testing Laboratory T0695

Test Report

Case no: 2018 - 210 02/03/2018

Determinand	Units	Methods used	South African National Standard (SANS) 241:2006&2015 for drinking water (partial) — Class 1 (Recommended levels) Class 2 (Maximum allowable for limited time) ** EU standard	Livestock Watering Recommended values	Dwaf specifications for irrigation	Client sample name: North Surface	NB 03 B (1) 02-03-18	NB 03 B (2) 02-03-18	NB 03 D (1) 02-03-18	NB 03 D (2) 02-03-18	NB 06 A 02-03-18	NB 06 B (1) 02-03-18	NB 06 B (2) 02-03-18	NB 07 (1) 02-03-18
						Lab number: 210-1	210-2	210-3	210-4	210-5	210-6	210-7	210-8	210-9
			Value	Value	Value	Value	Value	Value	Value	Value	Value	Value	Value	Value
**Chemical report**														
COD #	mg/L	Chem-TM04				92	97	64	26	192	72	51	22	726
Total organic carbon as C #	mg/L	Chem-TM24	≤ 10			28.1	18.0	16.7	8.37	7.81	11.5	13.1	12.4	83.0
Ammonia as N #	mg/L	Chem-TM18	≤ 1.5			0.26	<0.5	<0.5	1.58	3.83	2.81	<0.5	<0.5	0.55
pH #	pH units	Chem-TM06	5.5 tot 9.7		6.5 - 8.4	7.68	7.69	7.67	7.91	7.3	7.83	7.51	7.42	7.29
Electrical conductivity #	mS/m	Chem-TM06	≤ 170		<40	47.5	295	295	198	177	210	224	230	644
Calcium as Ca #	mg/L	Chem-TM02	≤150 - 300	≤1000		43	283	274	107	68	180	176	173	449
Magnesium as Mg #	mg/L	Chem-TM02	≤70 - 100	≤500		19	172	167	123	118	117	107	105	520
Sodium as Na #	mg/L	Chem-TM02	≤ 200	≤2000	0 - 70	28	268	261	168	158	169	222	219	351
Potassium as K #	mg/L	Chem-TM02	≤50 - 100			8.31	4.68	4.41	3.72	3.59	1.97	1.80	1.78	9.43
P-Alkalinity #	mg/L	Chem-TM06				0	0	0	0	0	0	0	0	0
M-Alkalinity #	mg/L	Chem-TM06				74.2	362	363	173	74.6	699	700	709	939
Fluoride as F #	mg/L	Chem-TM01	≤ 1.5	≤2		0.39	0.37	0.16	<0.1	<0.1	0.17	0.05	0.24	0.79
Chloride as Cl #	mg/L	Chem-TM01	≤ 300	≤1500	0 - 105	35	317	319	300	299	349	421	439	2190
Nitrite as N #	mg/L	Chem-TM01				<0.1	<0.1	<0.1	<0.1	<0.1	<0.1	<0.1	<0.1	<0.2
Bromide as Br #	mg/L	Chem-TM01	**≤3			0.50	1.85	2.01	2.13	1.91	1.71	2.27	1.93	11.33
Nitrate as N #	mg/L	Chem-TM01	≤ 11	≤22.5	0 - 5	<0.5	10.95	10.99	7.77	<0.5	<0.5	<0.5	<0.5	<1
Phosphate as PO_4 #	mg/L	Chem-TM01	*≤15.33			<1	<1	<1	<1	<1	<1	<1	<1	<2
Sulphate as SO_4 #	mg/L	Chem-TM01	≤ 500	≤1000		119	998	998	538	532	19.0	7.0	3.3	31.4
Calcium Hardness #	mg/L	calculated	≤375 - 750			108	708	685	268	171	449	441	432	1122
Magnesium Hardness #	mg/L	calculated	≤287 - 410			80	705	683	504	485	481	440	432	2131
Total Hardness as $CaCO_3$ #	mg/L	calculated	≤662 - 1160			188	1413	1369	772	656	930	881	864	3253
Total Dissolved Solids #	mg/L	calculated	≤ 1200			324	2453	2435	1450	1256	1537	1634	1648	4494
Sodium Adsorption Ratio(SAR) #		calculated			0 - 1.5	0.88	3.10	3.07	2.63	2.67	2.41	3.25	3.24	2.67
Aluminium as Al #	mg/L	Chem-TM02	≤ 0.300	≤5	0 - 0.5	0.093	<0.020	<0.020	<0.020	<0.020	<0.020	<0.020	<0.020	<0.020
Arsenic as As #	mg/L	Chem-TM02	≤ 0.010	≤1	0 - 0.1	<0.020	<0.020	<0.020	<0.020	<0.020	<0.020	<0.020	<0.020	<0.020
Barium as Ba #	mg/L	Chem-TM02	≤ 0.700			0.042	<0.020	<0.020	<0.020	<0.020	0.048	0.026	0.025	<0.020
Boron as B #	mg/L	Chem-TM02	≤ 2.400		0 - 0.5	0.036	0.220	0.222	0.121	0.089	0.082	0.098	0.086	0.169
Cadmium as Cd #	mg/L	Chem-TM02	≤ 0.003			<0.003	<0.003	<0.003	<0.003	<0.003	<0.003	<0.003	<0.003	<0.003
Cobalt as Co #	mg/L	Chem-TM02	≤ 0.500			<0.020	<0.020	<0.020	<0.020	<0.020	<0.020	<0.020	<0.020	<0.020
Chromium as Cr #	mg/L	Chem-TM02	≤ 0.050	≤0.05		<0.020	<0.020	<0.020	<0.020	<0.020	<0.020	<0.020	<0.020	<0.020
Copper as Cu #	mg/L	Chem-TM02	≤ 2.000	≤0.5	0 - 0.2	0.039	0.029	0.026	0.023	0.033	0.014	0.011	0.017	0.036
Iron as Fe #	mg/L	Chem-TM02	≤ 2.000 (chronic health)	≤10	0 - 0.5	0.159	0.097	0.014	0.036	0.043	0.008	0.007	0.068	0.109
	mg/L	Chem-TM02	≤ 0.300 (aesthetic)			<0.020	<0.020	<0.020	0.227	0.435	1.839	0.089	0.105	5.185
Manganese as Mn #	mg/L	Chem-TM02	≤ 0.400 (Chronic health)											
	mg/L	Chem-TM02	≤ 0.100 (Aesthetic)	≤10		<0.020	0.034	0.034	<0.020	<0.020	0.088	0.028	0.025	0.218
Nickel as Ni #	mg/L	Chem-TM02	≤ 0.070											
Molybdenum as Mo #	mg/L	Chem-TM02		≤0.01	0 - 0.02	<0.020	<0.020	<0.020	<0.020	<0.020	<0.020	<0.020	<0.020	<0.020
Lead as Pb #	mg/L	Chem-TM02	≤ 0.010	≤0.1	0 - 0.2	<0.015	<0.015	<0.015	<0.015	<0.015	<0.015	<0.015	<0.015	<0.015
Selenium as Se #	mg/L	Chem-TM02	≤ 0.040			<0.020	<0.020	<0.020	<0.020	<0.020	<0.020	<0.020	<0.020	<0.020
Vanadium as V #	mg/L	Chem-TM02	≤ 0.200			0.013	0.008	0.007	0.220	0.426	1.803	0.085	0.100	5.067
Zinc as Zn #	mg/L	Chem-TM02	≤ 5.000	≤20	0 - 1.0	0.020	<0.020	<0.020	<0.020	<0.020	<0.020	<0.020	<0.020	<0.020
**Bacterial report:**														
Total coliform	cfu/100ml	BAC-TM02	≤10	≤10	specifications will vary with the kind of crop irrigated.	>2420	>2420	>2420	99	74	22	488	517	>2420
E. coli	cfu/100ml	BAC-TM02	0	0		613	0	0	0	0	0	0	0	0

Note:

Results marked with (#) in this report, are not included in the SANAS Schedule of Accreditation for this laboratory.

Parameters higher than the specifications, are clearly **marked**. Tables with specifications included for comparison.

Bacteriological results obtained from samples older than the prescribed 24 hours may be inaccurate.

Signature: ___________________________

Dr L Deysel (Technical signatory / Technical manager)

END OF REPORT
